# Wood Ash Valorisation for Sustainable Materials: Circular Manufacturing, Characterization, Digital Modelling, and Industrial Applications

**DOI:** 10.3390/ma19142939

**Published:** 2026-07-08

**Authors:** Abrar Hussain, Himanshu S. Maurya, Oskars Leščinskis, Dmitri Goljandin, Maris Sinka, Xiangming Zhou, Ramin Rahmani, Jakob Kübarsepp, Tatjana Tambovceva, Diana Bajare

**Affiliations:** 1 Institute of Sustainable Building Materials and Engineering System, Riga Technical University, Paula Valdena iela 1, LV 1007 Riga, Latvia; oskars.lescinskis_1@rtu.lv (O.L.); maris.sinka@rtu.lv (M.S.); diana.bajare@rtu.lv (D.B.); 2 Department of Engineering Sciences and Mathematics, Luleå University of Technology, 97187 Luleå, Sweden; himanshudes@gmail.com; 3 Department of Mechanical and Industrial Engineering, Tallinn University of Technology, Ehitajate Tee 5, 19086 Tallinn, Estonia; dmitri.goljandin@taltech.ee (D.G.); jakob.kubarsepp@taltech.ee (J.K.); 4 Department of Civil and Environmental Engineering, Brunel University London, Uxbridge UB8 3PH, UK; xiangming.zhou@brunel.ac.uk; 5 CiTin—Centro de Interface Tecnológico Industrial, 4970-786 Arcos de Valdevez, Portugal; ramin.rahmani@ipvc.pt; 6 ProMetheus—Instituto Politécnico de Viana do Castelo (IPVC), 4900-347 Viana do Castelo, Portugal; 7 Institute of Governance and Security, Riga Technical University, Kalnciema iela 6-506, LV 1048 Riga, Latvia; tatjana.tambovceva@rtu.lv

**Keywords:** wood ash, additive manufacturing and recycling, cementitious materials, mechanical testing, computational analysis, sustainability

## Abstract

The increasing generation of wood ash (WA) from biomass combustion presents both an environmental challenge and an opportunity for sustainable resource utilization. This review provides a comprehensive assessment of recent advances in the valorization of WA for the development of sustainable engineering materials within a circular economy framework. Unlike previous studies that primarily focus on isolated applications of WA, this work integrates multiple technical dimensions, including material characterization, advanced manufacturing technologies, mechanical performance evaluation, computational modelling, and industrial commercialization pathways. Wood ash typically exhibits alkaline characteristics (pH 9–13.5) and particle sizes ranging from 1 to 1000 µm, enabling its application in a wide range of material systems. In cementitious materials, partial replacement of cement with WA (0.10–20%) generally improves mechanical performance, whereas excessive incorporation may reduce structural integrity. The high silica content (>62%) in certain WA types also enables its utilization in lightweight glass systems and radiation-shielding materials. Furthermore, WA has emerged as a promising functional filler in polymeric and ceramic composites, where additions above 0.5% can enhance dynamic mechanical properties and thermal stability. The review also examines standardized inspection and testing procedures, including quality control (QC) and quality assurance (QA) frameworks based on American Society for Testing and Materials (ASTM), Canadian Standards Association (CSA), and European standards, to ensure the reliability of WA-derived materials. Recent developments in artificial intelligence, machine learning, and computational modelling are highlighted for predicting mechanical behavior, optimizing processing parameters, and enabling digitalized manufacturing systems. In addition, circular manufacturing strategies and economic evaluation models, including break-even analysis, are discussed to assess the industrial feasibility of WA-based products. By integrating circular economy principles with materials engineering, digital technologies, and economic assessment, this review establishes a holistic framework for transforming wood ash from an industrial residue into value-added sustainable materials for construction, energy, and advanced composite applications.

## 1. Introduction

Wood-based biomass contributes significantly to the global renewable energy sector, accounting for approximately 55% of renewable energy production and nearly 6% of the total global energy supply. The combustion of approximately 3 gigatons (Gt) of biomass annually generates nearly 170 million tonnes (Mt) of wood ash (WA) worldwide. Reports indicate that the total global potential of biomass resources, including wood and its by-products, exceeds eight gigatons per year, producing nearly one thousand million tonnes of ash residues annually [1,2]. Consequently, WA has considerable potential as a secondary raw material for the production of sustainable engineering materials such as lightweight glass systems, nuclear radiation shielding composites, and cementitious materials [3,4,5,6]. During biomass combustion processes, two primary forms of ash are generated, namely wood bottom ash (WBA) and wood fly ash (WFA). In recent years, WBA has attracted increasing attention as a partial replacement for cement in construction materials due to its pozzolanic properties and mineral composition. Several industrial sectors, including thermal power plants, paper mills, and sawmills, produce large quantities of WBA, creating significant opportunities for its valorisation in sustainable construction technologies [7]. The concept of circularity, commonly referred to as the circular economy (CE), provides a promising pathway for the closed-loop utilization of WBA-based materials. Circular economy principles emphasize the reduction, reuse, and recycling of industrial residues to minimize environmental impacts and promote resource efficiency. The integration of WBA into manufacturing processes can therefore reduce waste generation and contribute to achieving the Pan-European 2050 net-zero carbon emission target. However, despite these advantages, the implementation of circular economy strategies in manufacturing industries still faces several operational, technological, and engineering challenges that require further research and innovation [8,9,10,11,12].

**Figure 1:**
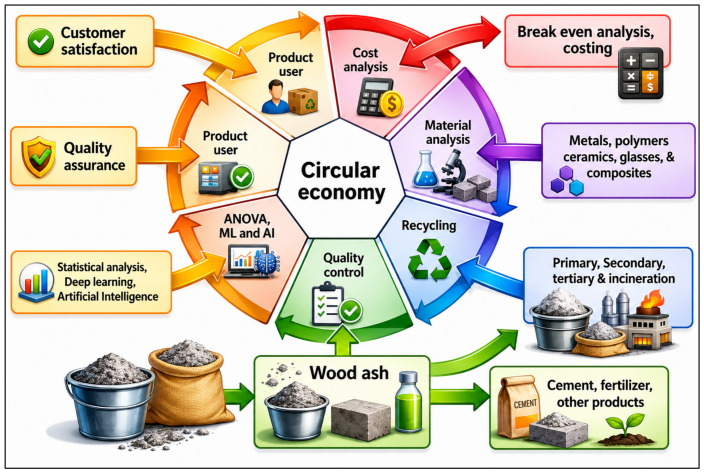
The circularity model for sustainable recycling and additive manufacturing [8]. Generated by ChatGPT (OpenAI, GPT-5.5).

Hussain et al. proposed a circularity model for sustainable industrial manufacturing, which integrates material analysis, recycling strategies, and economic evaluation within a circular economy framework [8]. A modified version of the circular economy (CE) model is illustrated in Figure 1. The model comprises several interconnected stages, including WBA analysis, recycling through additive manufacturing, quality control, computational analysis, quality assurance, final product development, and cost evaluation. The initial analysis of WBA ensures the suitability of the material and supports the selection of appropriate manufacturing processes [8,13,14]. Careful selection of processing techniques is essential for improving the sustainability and performance of the final products. Within this framework, CE incorporates technical management strategies, advanced manufacturing technologies, and systematic quality control procedures involving both mechanical and chemical investigations [15]. Restorative, regenerative, and closed-loop manufacturing principles associated with CE help address complex challenges related to resource utilization and sustainable production. Quality control and quality assurance procedures are employed to evaluate the physical, chemical, and mechanical properties of manufactured materials, allowing the assessment of performance and service life during the investigation stage [16,17,18]. In recent years, computational analysis and machine learning techniques have emerged as powerful tools for predicting the behaviour and potential industrial applications of manufactured materials. Software platforms such as MATLAB and Python-based analytical tools are commonly used to model material behaviour, optimize processing parameters, and support automation in manufacturing systems [17,18]. Finally, the manufactured products are standardized according to industrial requirements and customer satisfaction. Economic analysis, including evaluation of fixed, variable, labour, material, and manufacturing costs, is incorporated to assess the feasibility of production systems [8]. Among the various technical components of the circularity model, the selection of appropriate manufacturing processes remains a fundamental factor in achieving environmentally sustainable and resource-efficient production [4,8,11,13,18,19]. This integrated circularity framework provides a systematic pathway for transforming wood ash waste into value-added engineering materials while minimizing environmental impacts. Such models are essential for advancing sustainable manufacturing systems and supporting industrial implementation of circular economy principles [20,21].

Additive manufacturing, commonly referred to as three-dimensional printing (3DP), together with recycling technologies, has emerged as an innovative approach to implementing circular economy principles in modern processing industries [8,22]. This technology enables the fabrication of complex and customized products using a wide range of materials including metals [23], glass [24], polymers [25], ceramics [26], and biomaterials [27]. Consequently, additive manufacturing has been widely applied in several industrial sectors such as aerospace [28], electronics [29], construction [30], and medical engineering [28]. In the context of sustainable materials development, 3DP offers significant potential for transforming wood ash (WA) into value-added products such as concrete structures, nuclear radiation shielding composites, and lightweight glass materials [3,4,5,6]. Furthermore, the incorporation of fibre reinforcements, additives, fillers, binders, and hybrid mixtures can significantly enhance the mechanical and functional properties of WA-based materials [31,32]. The performance of additively manufactured products strongly depends on processing parameters such as nozzle design, printing speed, pressure, temperature, particle morphology, fluidity, setting time, and particle size distribution [31,32,33]. Several studies have shown that additive manufacturing can utilize up to 60% of waste materials while reducing labour costs by approximately 80% and execution time by nearly 70% compared with conventional manufacturing methods [34]. Despite these advantages, the rapid growth of additive manufacturing and industrial recycling technologies requires further innovation in the testing and characterization of WA-based materials. The incorporation of WA and other waste materials can significantly influence the mechanical behaviour of concrete and composite systems. In extrusion-based 3DP processes, the layer-by-layer deposition mechanism may reduce structural strength and introduce microstructural defects. In particular, the formation of porosity, voids, and micro-cracks in printed components necessitates the development of advanced inspection and testing techniques to ensure the reliability and performance of WA-based materials [16,35,36,37,38].

Physical and chemical investigations play a crucial role in determining the performance and reliability of wood ash (WA)-based novel materials. These investigations facilitate the transition of laboratory-scale pilot studies into commercial-scale production by evaluating the structural integrity and functional properties of the developed materials. Mechanical testing methods such as compressive strength, flexural strength, and split tensile strength tests are widely employed to assess the usability, suitability, and service life of WA-based materials for industrial applications [36,37,38,39,40,41]. For instance, Li et al. utilized several standard testing procedures including American Society for Testing and Materials (ASTM) D3043 for bending, ASTM D3501 for compression, and European standards (EN) 323 and EN 317 for water absorption in the evaluation of WA-based concrete systems [42]. In addition to mechanical testing, thermal analysis techniques are often introduced to investigate the thermal stability and degradation behaviour of WA-derived materials. Differential scanning calorimetry (DSC) and thermogravimetric analysis (TGA) are commonly used to determine important thermal parameters such as decomposition temperature, phase transitions, and thermal stability [43]. Furthermore, advanced characterization techniques including scanning electron microscopy (SEM), spectroscopy, and other surface evaluation methods are applied to examine cross-sectional morphology, microstructural features, and defect formation within the materials [43]. In recent years, computational analysis, artificial intelligence (AI), and machine learning approaches have increasingly been applied to predict the behaviour and performance of WA-based materials [21]. These computational tools enable the prediction of mechanical properties, optimization of processing parameters, and identification of potential industrial applications. In addition, software-based modelling and algorithm development can support automation and digitalization in modern manufacturing systems [21,44,45]. Besides computational modelling, statistical techniques such as analysis of variance (ANOVA) are frequently used to evaluate the influence and significance of manufacturing parameters on the mechanical behaviour and performance of WA-based materials [4,13,46,47,48,49].

Computational technologies, machine learning techniques, artificial intelligence (AI), and digitalization play an increasingly important role in modernizing commercial additive manufacturing of wood ash (WA)-based materials [19,50,51,52]. The implementation of advanced computing tools provides an engineering pathway for integrating circular economy principles with digital manufacturing systems. As a result, circular production strategies and green manufacturing practices can be achieved through optimized processing, improved resource efficiency, and enhanced product design. Despite these advantages, the utilization of WA in additive manufacturing presents several technical challenges related to material variability, process control, and structural reliability. Pilot-scale production of WA-based materials is therefore essential for identifying processing limitations and developing innovative solutions for industrial implementation [53,54,55,56,57,58]. As illustrated in Figure 1, the proposed circularity model integrates testing procedures, quality assurance mechanisms, and customer satisfaction criteria to standardize the final products and ensure their industrial applicability. Cost evaluation represents the final stage of the manufacturing framework. In this context, break-even analysis (BEA) is widely used as an effective industrial tool for predicting the economic feasibility of manufacturing processes and determining the relationship between production cost, revenue, and profit [8]. Cost assessment typically includes fixed, variable, material, labour, and operational costs associated with starting, operating, expanding, or reducing production during the implementation of circular economy strategies [8,9]. Overall, the integrated consideration of WA characterization, additive manufacturing or recycling processes, chemical and physical testing, computational modelling, quality control, quality assurance, and customer satisfaction forms the core technical paradigm of the circular economy framework [8,59]. The application of these interconnected approaches can significantly enhance sustainable manufacturing, promote circular industrial production, and support the development of environmentally responsible engineering materials, as illustrated in Figure 1 [8,60]. Therefore, integrating digital technologies, circular economy strategies, and economic assessment models is essential for the successful industrial implementation of WA-based sustainable manufacturing systems.

**Figure 2:**
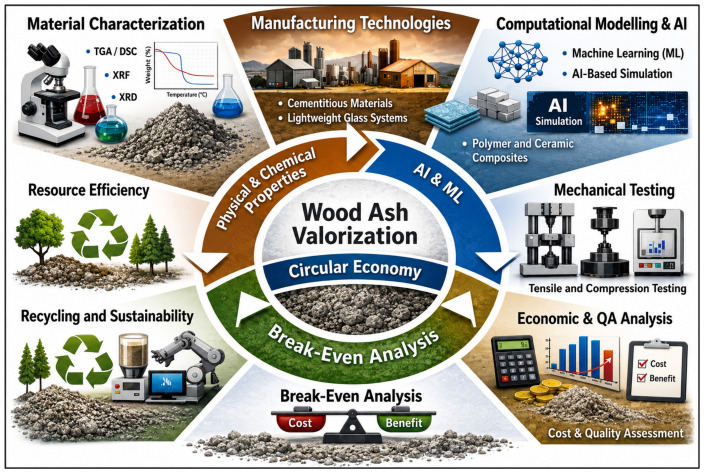
Circular economy framework for wood ash (WA) valorization, integrating characterization, manufacturing technologies, AI modelling, mechanical testing, and economic analysis. Generated by ChatGPT (OpenAI, GPT-5.5).

The present review provides a comprehensive analysis of recent developments in the valorisation of wood ash (WA) for sustainable material production within a circular economy framework. Unlike previous studies that primarily focus on individual applications of WA, this review integrates multiple technical dimensions, including material characterization, manufacturing technologies, mechanical testing, computational modelling, and industrial commercialization pathways. The review systematically examines the physical, chemical, thermal, and spectroscopic characteristics of WA to evaluate its suitability for recycling and advanced manufacturing processes. Particular attention is given to the production of cementitious materials, lightweight glass systems, and polymeric or ceramic composites derived from WA. Furthermore, recent advances in computational approaches, including machine learning and artificial intelligence, are discussed to highlight their potential for predicting mechanical performance, optimizing processing parameters, and enabling digitalized manufacturing systems. In addition, quality control and quality assurance strategies are examined together with economic feasibility assessments through cost modelling and break-even analysis. By integrating circular economy principles with engineering design, materials processing, and industrial evaluation, this review establishes a holistic framework for advancing the sustainable manufacturing and commercialization of wood ash-based materials. This integrated perspective aims to support future research and industrial adoption of WA-derived materials while contributing to resource efficiency, waste reduction, and environmentally sustainable manufacturing systems (see Figure 2).

To ensure a comprehensive and systematic review, the literature related to wood ash (WA) valorization and sustainable materials was collected from major scientific databases, including Scopus, Web of Science, ScienceDirect, SpringerLink, Wiley Online Library, and Google Scholar. Searches were performed using combinations of the keywords “wood ash”, “wood bottom ash”, “wood fly ash”, “biomass ash”, “circular economy”, “waste valorization”, “additive manufacturing”, “3D printing”, “cementitious materials”, “glass materials”, “polymer composites”, “ceramic composites”, “mechanical properties”, “thermal analysis”, “artificial intelligence”, “machine learning”, “computational modelling”, and “economic analysis”. Boolean operators (AND and OR) were used to refine the search strategy. The review mainly covers publications from 2000 to 2025, with emphasis on recent studies published during 2015–2025. Peer-reviewed journal articles were prioritized, while selected conference proceedings, standards, books, and technical reports were included when they provided essential information. Duplicate records and studies outside the scope of wood ash characterization, processing, manufacturing, digital modelling, and industrial applications were excluded. The final selection of literature was based on relevance, scientific quality, and contribution to the development of sustainable wood ash-derived materials.

## 2. Analysis of Wood Ash and Its Types

The combustion of wood biomass produces two primary ash fractions, namely WBA and wood fly ash (WFA). Collectively referred to as WA, these residues consist of both organic remnants and inorganic mineral phases generated during the thermochemical decomposition of lignocellulosic biomass. The particle size distribution of WA varies depending on the combustion process, where the average particle sizes of WBA **and** WFA are typically below 261 μm and 35 μm, respectively, indicating the significantly finer nature of fly ash particles [59,61,62,63,64]. Table 1 presents the representative chemical composition of WBA. Morphological observations reveal that WA particles commonly exhibit angular, spherical, and irregular geometries, reflecting the heterogeneous transformation of mineral components during high-temperature combustion. Chemically, WA is composed mainly of metal oxides such as SiO_2_, CaO, Al_2_O_3_, Fe_2_O_3_, K_2_O, and MgO, along with trace concentrations of heavy metals as summarized in Table 1 [65,66,67,68,69,70,71]. Combustion of wood in an oxygen-rich environment promotes oxidation of organic constituents and mineral elements, leading to the formation of highly alkaline ash with elevated pH values. The simplified oxidation reaction representing wood biomass combustion can be expressed as:

Wood + O_2_ → Charcoal + CaO + CaCO_3_ + CO_2_ + H_2_O

CaO + H_2_O ↔ Ca (OH)_2_

Ca (OH)_2_ + CO_2_ ↔ CaCO_3_ + H_2_O

CaCO_3_ + H_2_O+ CO_2_ ↔ Ca (HCO_3_)_2_

where wood represents the generalized empirical formula of lignocellulosic biomass. The residual ash contains the inorganic mineral constituents that remain after the volatilization of organic components.

The potential of hydrogen (pH) of wood ash (WA) typically ranges between 9 and 13.5, reflecting its strongly alkaline character and significant mineral content [72]. Etiegni and Campbell [68] reported that the yield of WA decreases by approximately 45% as the combustion temperature increases from 538 to 1093 °C, primarily due to volatilization and thermal decomposition of mineral phases. Their investigation further indicated that WA particles exhibit an average particle size of approximately 230 μm, although the particle morphology and size distribution may vary depending on combustion and collection conditions. Increasing combustion temperature also promotes the enrichment of metallic and heavy metal constituents in the residual ash, as volatile components are progressively removed during thermal treatment. In contrast, the behaviour of alkali and alkaline earth metals, particularly sodium (Na) and potassium (K), is strongly temperature-dependent. At temperatures below 500 °C, mineral phases are mainly present as carbonates and bicarbonates, whereas oxide phases dominate at temperatures exceeding 1000 °C due to thermal decomposition reactions [73,74,75]. Overall, the chemical and mineralogical composition of WA is highly heterogeneous and is influenced by several parameters including wood species, combustion technology, operating temperature, ash collection system, and biomass origin [70,71,72,73,74,75]. A summary of the typical chemical composition of WA reported in the literature is presented in Table 1.

**Table 1:** Chemical composition of wood ash.

Ash Type	Percentage of Oxides (wt.%)											References
	SiO_2_	Al_2_O_3_	Fe_2_O_3_	CaO	MgO	Na_2_O	K_2_O	SO_3_	TiO_2_	F_2_O_2_	LOI	
WBA	53	13	6	13	3	5	7	-	-	-	9	[62]
WBA	9	-	9	59	-	-	12	-	-	-	59	[9,59]
WBA	`21	`3	1.50	48	7	-	`-	-	-	-	-	[41,70]
WA	49	11	1	12	5	9	5.50	-	-	-	9	[71]
WBA	69	8	5.50	11	3	0.50	6	1	-	-	15	[74]
WBA	7	3	1	32	2	-	12	0.10	-	-	42	[76]
WA	40	11	4	16	4	1	5	1	1	1	8	[77]
WFA	1.5	1.0	-	40.9	7.1	-	14.3	-	-	0.6	-	[65]
WFA	6.1	2.0	-	46.10	4.0	-	23.4	-	-	0.7	-	[65]
WFA	12.4	0.1	-	68.2	11.5	-	2.6	-	-	1.1	-	[65]
WFA	1.9	0.6	-	77.3	2.4	-	8.9	-	-	0.7	-	[65]
WFA	4.5	-	-	83.5	2.5	-	5.5	-	-	0.4	-	[65]
WFA	-	-	-	57.7	10.9	-	9.3	-	-	-	-	[65]
WFA	4.6	0.9	-	47.6	3.5	-	12.0	-	-	2.7	-	[64]
WFA	5–15	1–8		30–60	2–10		5–20			1–5		[2]
WFA	3–10	1–5		25–50	2–8		5–15			1–3		[63]
WFA	5.20	1–10		20–45	2–10		5–15			1–5		[45,77]

### 2.1. Physical Analysis of Wood Ash

**Figure 3:**
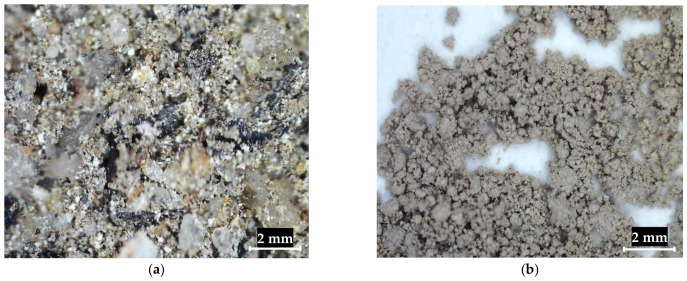

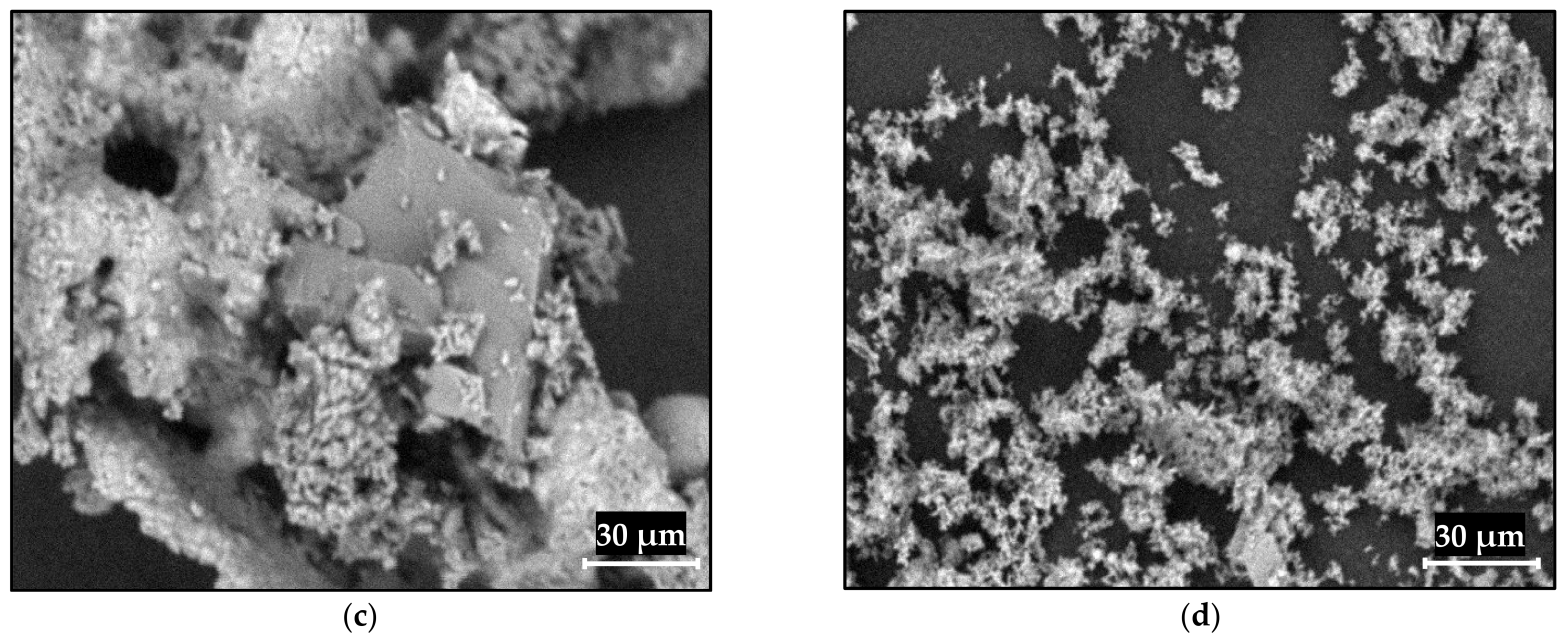
Digital and SEM images of WBA and WFA: (**a**) digital image of WBA, (**b**) digital image of WFA, (**c**) SEM image of WBA with angular-shaped particles, and (**d**) SEM image of WFA with spherical-shaped particles.

Wood ash (WA) is primarily composed of major mineral oxides including silica (SiO_2_), alumina (Al_2_O_3_), iron oxide (Fe_2_O_3_), magnesium oxide (MgO), calcium oxide (CaO), sodium oxide (Na_2_O), and potassium oxide (K_2_O), which originate from the inorganic constituents of biomass and the thermal transformations occurring during combustion. These mineral phases largely determine the physicochemical behavior and potential reutilization pathways of WA in construction, environmental, and composite material applications. Figure 3a,b illustrate the scanning electron microscopy (SEM) micrographs of wood bottom ash (WBA) and wood fly ash (WFA), respectively. As shown in Figure 3c,d, the microstructure of WBA and WFA is predominantly amorphous, reflecting rapid thermal reactions and partial melting during biomass combustion.

WBA particles generally exhibit angular and heterogeneous morphologies, whereas WFA particles tend to display smoother and more spherical structures, which are typically formed through melting, vaporization, and subsequent condensation of mineral phases within the flue gas stream. Nevertheless, both ash fractions frequently contain irregularly shaped particles due to the complex physicochemical transformations occurring during combustion and ash deposition.

The particle size distribution of WA varies considerably depending on combustion technology, biomass species, operating temperature, and ash collection systems. Previous studies report that WBA particles typically range from 10 to 1000 μm, while WFA particles are substantially finer, generally ranging from 0.2 to 100 μm [73,74,75,76]. In addition to particle morphology, several physicochemical properties strongly influence the reactivity and engineering performance of WA-derived materials. The reported mean values of specific surface area, particle size, density, and pH for WBA generally fall within the ranges of 0.80–1.8 m^2^ g^−1^, 100–300 μm, 2.6–2.9 g cm^−3^, and 11.75–12.50, respectively. In comparison, WFA typically exhibits a lower specific surface area but finer particle size, with reported ranges of 0.05–0.85 m^2^ g^−1^, 10–140 μm, 2.4–2.5 g cm^−3^, and 12.70–13.75, respectively [76,77,78,79,80,81,82]. These physicochemical characteristics, particularly particle morphology, surface area, and alkalinity, play a crucial role in determining the suitability of WA for applications such as cementitious systems, glass–ceramic production, soil stabilization, and polymer or ceramic composites.

### 2.2. Chemical Analysis of Wood Ash

Silica (SiO_2_), alumina (Al_2_O_3_), calcium oxide (CaO), and iron oxide (Fe_2_O_3_) are the major constituents of wood ash (WA) and play a critical role in determining its pozzolanic and binder properties. According to EN 450-1, the combined content of the principal pozzolanic oxides (SiO_2_ + Al_2_O_3_ + Fe_2_O_3_) should exceed 70% to ensure favorable reactivity and performance in cementitious systems [80]. Silica contributes significantly to the alkalinity and rheological behaviour of concrete mixtures and can influence workability and flow characteristics. In WA, silica may exist in both amorphous and crystalline phases, which strongly affects its pozzolanic reactivity [81,82].

Medina et al. analyzed 21 wood ash samples, including both WBA and WFA collected from different biomass thermal power plants, and reported the following compositional distribution: CaO (48%) > SiO_2_ (13%) > K_2_O (10%) > MgO (5%) > Al_2_O_3_ (3%) > P_2_O_5_ (3%) [83]. The presence of higher concentrations of P_2_O_5_ can retard the hydration process and delay the setting time of concrete. Similarly, elevated levels of alkali oxides (Na_2_O and K_2_O) may promote alkali–aggregate reactions, leading to the formation of reactive concrete aggregates and consequently reducing the durability of cementitious materials. Excessive SO_3_ content can also induce chemical attack in concrete systems, resulting in deterioration of microstructure and reduced service life of WA-based materials [67,84]. Furthermore, oxides such as CaO and MgO significantly contribute to the alkaline nature of WA, collectively increasing the pH of the material, which can reach values close to 12. The dominant mineral phases typically identified in wood ash include free CaO, MgO, dicalcium silicate (2CaO·SiO_2_), and CaCO_3_, which play an important role in governing the chemical reactivity and binding behaviour of WA-derived materials [73,84,85].

### 2.3. Thermal Analysis of Wood Ash

Combustion temperature plays a critical role in determining the physicochemical composition and properties of wood ash (WA). Variations in combustion temperature significantly influence parameters such as loss on ignition (LOI), density, moisture content, and mechanical characteristics of WA [7,86]. Higher combustion temperatures generally promote more complete oxidation of organic constituents in wood, resulting in lower LOI values and increased mineral stability in the residual ash. Thermal characterization techniques such as thermogravimetric analysis (TGA) and differential scanning calorimetry (DSC) indicate that the thermal decomposition and combustion of wood materials typically occur within the temperature range of 550–1250 °C [1,7,87,88]. These thermal processes govern the transformation of biomass into mineral-rich ash phases, thereby influencing the chemical composition and potential applications of WA in cementitious materials, glass systems, and composite manufacturing. Wood biomass ash is typically calcium-rich, with CaO usually being the dominant oxide, followed by SiO_2_ and K_2_O, while MgO, Al_2_O_3_, and Fe_2_O_3_ occur in smaller quantities. The composition of wood fly ash strongly depends on biomass type (hardwood/softwood), combustion temperature, and ash collection system.

## 3. Recycling and Additive Manufacturing of Wood Ash Novel Materials

**Figure 4:**
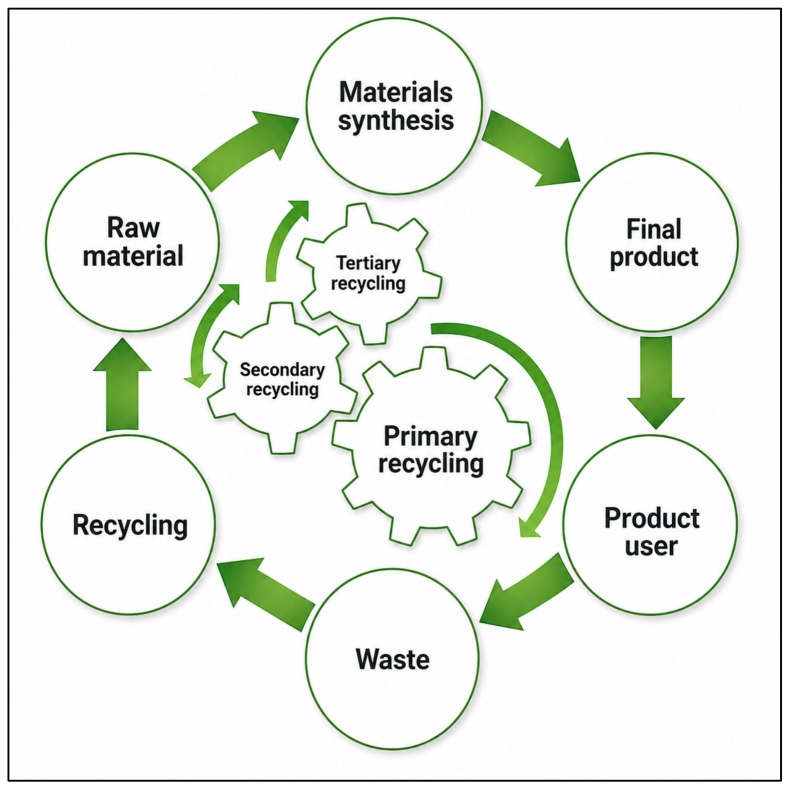
Three-dimensional printing schemes of wood ash.

Additive manufacturing (AM), also known as three-dimensional printing (3DP), has recently gained significant attention for the recycling and valorisation of wood ash (WA)-based materials due to its advantages in process automation, cost efficiency, and environmentally sustainable production [89], as illustrated in Figure 4. The proposed 3DP strategy for WA-based materials can be implemented through two principal processing schemes. In the first scheme, relatively high-purity WA (typically >99% mineral content, as summarized in Table 1) can be directly utilized as a raw or recycled feedstock for the fabrication of cementitious concretes, lightweight glass systems, and nuclear shielding composites. In this approach, the content of pozzolanic oxides such as SiO_2_, Al_2_O_3_, and Fe_2_O_3_ serves as a key compositional parameter governing the reactivity and structural performance of WA-based concrete materials. In the second scheme, additional pre-treatment and purification processes are applied to enhance the quality and compositional uniformity of WA. These purification steps enable the selective recovery of valuable mineral phases and improve the suitability of WA for advanced manufacturing routes, including high-precision additive manufacturing applications [89,90,91,92,93,94,95,96].

Concrete represents one of the most prominent application areas for wood ash (WA)-derived materials, owing to the pozzolanic and mineralogical characteristics of WA. Hamid et al. investigated the incorporation of WA obtained from restaurants and wood-processing plants by replacing cement at 0, 10, 15, 20, and 25 wt.% in concrete mixtures. Their results demonstrated that a 10 wt.% partial replacement of cement with WA yielded the highest compressive strength after 7 and 28 days of curing [65]. Similarly, Thomas et al. evaluated the effect of wood fly ash (WFA) on concrete performance by replacing cement within the range of 0–100 wt.%, while also incorporating blast furnace slag as a supplementary material. Their study reported optimum mechanical performance at 50 wt.% WFA replacement, highlighting the synergistic interaction between WFA and slag in the cementitious matrix [97,98,99]. Furthermore, the performance of WA-based concrete is strongly influenced by the CaO content present in the ash. According to the Canadian Standards Association (CSA) A23.5 and ASTM C618, WA can be categorized into three classes based on CaO concentration: Type F (CaO ≤ 8%), Type CI (CaO ≤ 20%), and Type CH (CaO > 20%) [98]. Malaiškienė et al. also investigated cement substitution by incorporating 15% WBA and 15% silica fly ash (total 30% replacement), achieving satisfactory compressive strength results with only a 6% reduction compared with conventional concrete [100]. In addition, the physicochemical nature and particle morphology of ash significantly influence its suitability for additive manufacturing (AM) of concrete-based materials [101]. Overall, numerous studies indicate that the incorporation of approximately 10–20% WA in cementitious systems can enhance or maintain the mechanical performance of concrete while promoting sustainable material utilization [54,58]. Although WA can partially replace cement, excessive incorporation may adversely affect workability, hydration kinetics, and long-term strength. Consequently, practical replacement levels are generally limited to approximately 10–20 wt.% depending on ash composition and curing conditions.

The replacement ratio of wood ash (WA) as a partial substitute for cement plays a critical role in determining the mechanical performance of concrete. Several studies have reported that incorporating approximately 5% WA can enhance the compressive strength (CS) of concrete mixtures due to improved pozzolanic reactivity and particle packing effects [16,36,102]. In some cases, even very small substitutions (0.5–2%) of wood bottom ash (WBA) or wood fly ash (WFA) have been shown to improve compressive strength and microstructural stability [103,104]. Cheah et al. demonstrated that the incorporation of 8% WA significantly improved the overall mechanical properties of concrete, with compressive strength values ranging from 35 to 54 MPa after 3, 7, and 28 days of curing [105]. Many studies further indicate that a 10–15% replacement of cement with WA generally produces optimal mechanical performance while maintaining satisfactory durability and structural integrity [106,107,108]. In some cases, even 20% WA substitution has been reported to maintain comparable water absorption, density, and strength characteristics of concrete [109]. However, excessive replacement levels typically lead to a decline in mechanical performance and material quality. Higher WA contents may introduce microstructural defects, including increased porosity, voids, and internal microcracks, which promote stress concentration and strain localization within the cementitious matrix [110,111,112]. These structural irregularities ultimately reduce the overall strength and durability of WA-based materials.

The production of glass systems from ash-derived materials remains a relatively underexplored research area, despite its promising potential for advanced functional applications. Several studies have demonstrated that ash-based glass materials exhibit effective shielding capabilities against particle and ionizing radiation, highlighting their potential use in radiation protection systems. Consequently, various types of ash can be utilized as alternative silica sources for the manufacturing of glass and glass–ceramic systems. However, for efficient glass formation, the SiO_2_ content in agricultural or biomass ashes should generally exceed approximately 62%, ensuring sufficient network formation in the glass matrix. Furthermore, the incorporation (doping) of heavy metal oxides such as lead (Pb), bismuth (Bi), and tungsten (W) into these glass systems significantly enhances their gamma-ray shielding efficiency and overall protective performance. The presence of these high atomic-number elements increases photon attenuation, thereby improving radiation absorption capacity. Several recent investigations have reported the successful synthesis and characterization of ash-derived radiation-shielding glass systems, demonstrating their potential for applications in nuclear infrastructure, medical radiation facilities, and protective materials [113,114,115]. High-silica WA is particularly suitable for glass production; however, excessive alkali content and compositional variability may influence viscosity, crystallization behavior, and thermal stability, thereby restricting processing windows.

**Figure 5:**
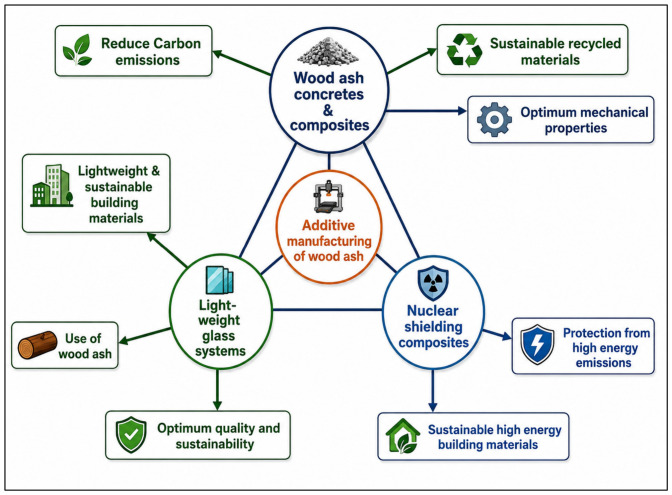
Proposed commercial applications of wood ash. Generated by ChatGPT (OpenAI, GPT-5.5).

In recent years, wood-derived materials and wood ash (WA) have attracted increasing attention for the fabrication of polymer- and glass-based composites, where WA is commonly utilized as a functional filler or reinforcing phase. The incorporation of wood residues, WA, and their derived products into composite systems has demonstrated significant potential for enhancing the thermal stability, mechanical performance, and radiation shielding capabilities of advanced materials [116,117,118,119], as illustrated in Figure 5. The mineral-rich composition of WA, particularly its silica and metal oxide content, contributes to improved interfacial bonding, structural rigidity, and radiation attenuation properties when incorporated into polymeric and glass matrices. Consequently, WA-based fillers are increasingly being explored as sustainable and cost-effective alternatives for the development of high-performance composite materials. The incorporation of ash-derived fillers into polymer matrices has emerged as a promising strategy for developing sustainable and high-performance composite materials. Among various biomass residues, wood ash (WA) has attracted considerable attention due to its abundance, low cost, and mineral-rich composition, which typically includes silica, calcium oxide, alumina, and other metal oxides [116,120,121,122]. These inorganic constituents enable WA to function as an effective reinforcing filler in polymer composites, improving mechanical strength, stiffness, and thermal stability while simultaneously reducing material cost and environmental impact. Several studies have reported the successful fabrication of epoxy, polyester, and thermoplastic-based composites reinforced with wood ash particles, demonstrating improvements in tensile strength, hardness, wear resistance, and dimensional stability. In addition to mechanical enhancement, WA-filled polymer composites also exhibit improved thermal resistance and radiation shielding performance, primarily due to the presence of high-density mineral phases within the ash. The particle morphology and chemical composition of WA can further influence interfacial bonding between the filler and polymer matrix, thereby affecting the overall composite performance. Moreover, surface treatment and particle size optimization of WA fillers have been shown to significantly improve dispersion and compatibility with polymer matrices. From a sustainability perspective, the utilization of WA as a functional filler contributes to waste valorisation and circular economy strategies, enabling the transformation of biomass combustion residues into value-added engineering materials. Consequently, polymer–wood ash composites represent a promising class of eco-friendly materials for structural, thermal insulation, and radiation shielding applications, particularly in construction, energy infrastructure, and protective systems [123,124,125]. In composite systems, low-to-moderate WA contents generally improve stiffness and thermal stability, whereas excessive loading may reduce interfacial bonding, processability, and toughness. Therefore, optimization of filler content and particle morphology remains critical for industrial applications.

Although wood ash exhibits considerable potential for sustainable material development, its suitability strongly depends on the intended application and the physicochemical characteristics of the ash. In cementitious systems, WA replacement levels below approximately 10–20 wt.% generally provide acceptable mechanical performance, whereas excessive incorporation may adversely affect workability and strength development. High-silica WA is particularly suitable for lightweight glass and ceramic applications, while polymeric and hybrid composites benefit from low-to-moderate filler contents to maintain adequate interfacial bonding and processability. Material variability, alkali content, porosity, moisture sensitivity, and long-term durability remain important limitations that constrain broader industrial adoption. Consequently, the selection of WA-derived materials should be application-specific and supported by standardized characterization, quality control procedures, and techno-economic assessment. From an industrial perspective, the performance boundaries of WA-based materials are determined not only by mechanical properties but also by manufacturability, cost-effectiveness, environmental benefits, and regulatory requirements [126,127,128].

The suitability of wood ash (WA) for different industrial applications depends strongly on its chemical composition, particle size distribution, mineralogical characteristics, and processing route. Although WA has demonstrated considerable potential in cementitious materials, lightweight glass systems, and polymer or ceramic composites, the optimum incorporation level and performance requirements vary significantly depending on the intended application. In cementitious systems, moderate WA replacement levels generally provide acceptable mechanical properties, whereas excessive contents may reduce workability and strength. Similarly, high-silica WA is advantageous for glass and ceramic applications, while polymer composites require careful control of filler content to maintain adequate interfacial adhesion and processability. Therefore, application-specific optimization, quality control, and standardization are essential for successful industrial implementation [129,130,131].

## 4. Inspection and Testing of Wood Ash Novel Materials

The utilization of wood ash (WA) significantly influences the mechanical properties, performance, and long-term durability of the manufactured materials in which it is incorporated. The effects of WA depend on several factors, including its chemical composition, particle morphology, replacement ratio, and processing conditions, which collectively determine the structural behaviour of WA-based products. Therefore, the selection of appropriate inspection, characterization, and testing methods is essential for accurately evaluating the quality, reliability, and performance of WA-derived materials. These evaluation techniques must be carefully chosen according to the intended application and material system, such as cementitious composites, glass systems, or polymer-based materials [132].

**Figure 6:**
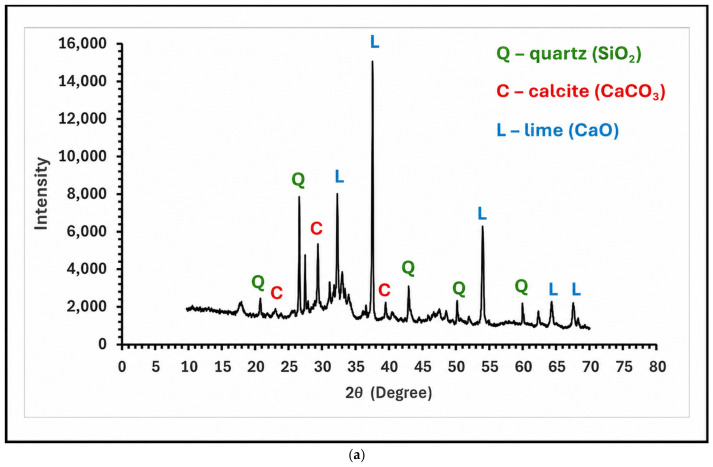

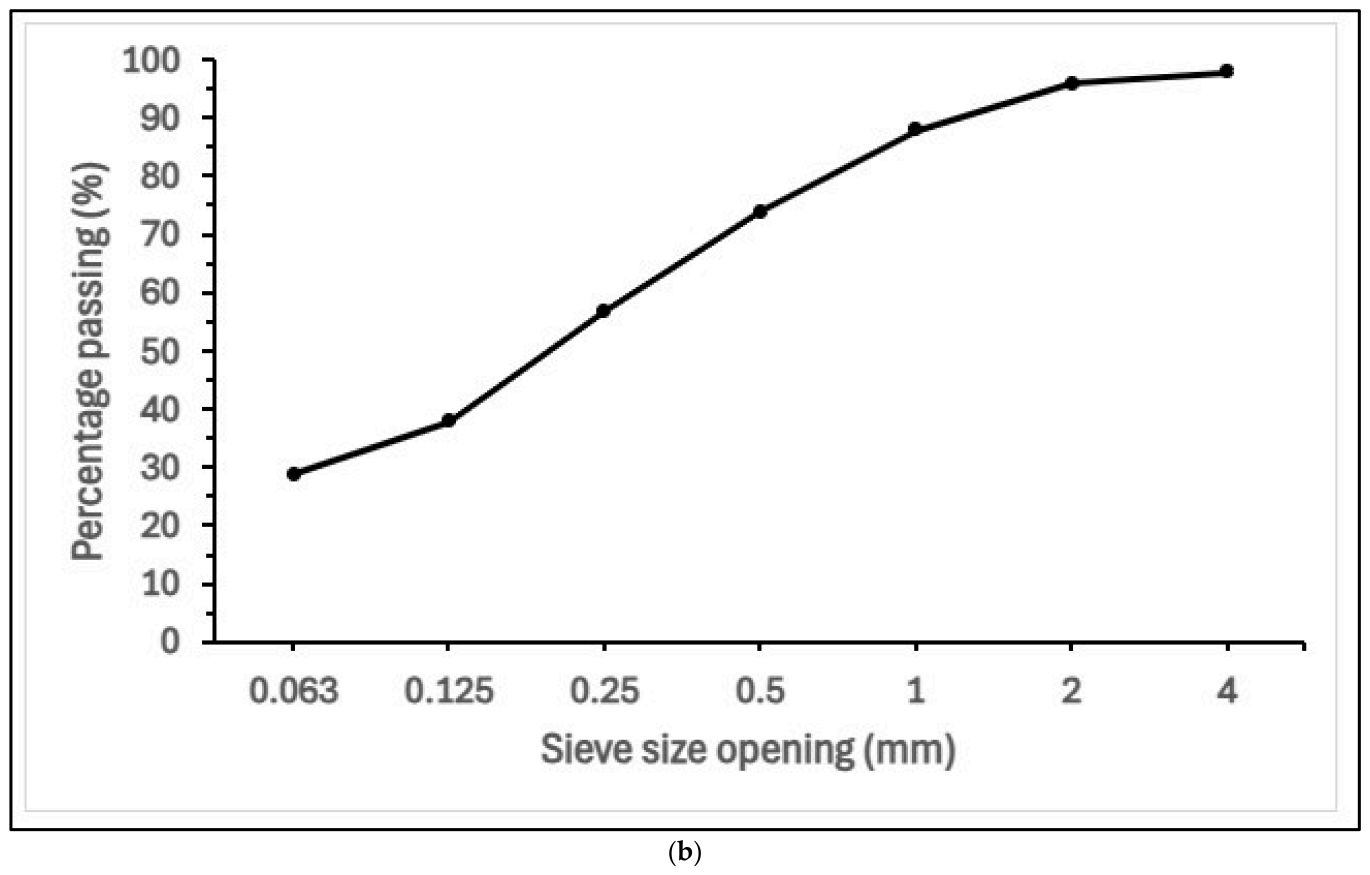
Representative demonstration of (**a**) X-ray diffraction and (**b**) particle size distribution of wood ash.

The physical and microstructural characteristics of wood ash (WA), including wood bottom ash (WBA) and wood fly ash (WFA), play an important role in determining the performance of WA-based materials. The particle morphology and size distribution of WA can be examined using scanning electron microscopy (SEM) (Figure 6a). At the microscale, WA particles typically exhibit irregular shapes, porous surfaces, and a broad particle size distribution, often accompanied by partially amorphous structures, which influence the reactivity and packing behaviour of the ash particles [102]. Chemically, WA is primarily composed of metal oxides such as Al_2_O_3_, SiO_2_, Fe_2_O_3_, MgO, CaO, Na_2_O, and K_2_O, which significantly contribute to its cementitious and pozzolanic properties [133]. The mineralogical composition of WA can be further identified using X-ray diffraction (XRD) analysis (Figure 6b), which enables the detection of crystalline phases associated with these oxides. In addition to the major components, trace oxides such as TiO_2_, P_2_O_5_, SO_3_, ZnO, SrO, ZrO_2_, and BaO are also present in smaller quantities [59,62,64,67,68,69,70,71,72,73,74,75,76,134]. The alkaline nature of WA is another critical parameter affecting its compatibility with construction and advanced materials. Typically, WA exhibits pH values in the range of 10–13.5, which is favourable for applications in cementitious systems and other engineered materials. Furthermore, the specific surface area of WA generally ranges from 2835 to 7810 cm^2^ g^−1^, contributing to its high reactivity and particle interaction within composite matrices [62,135]. According to Carević et al. and other researchers, WBA particles exhibit a size distribution ranging from approximately 10 to 2000 μm. This particle size range, which lies between that of cement and sand, can improve the packing density, rheological behaviour, and overall microstructural compactness of WA-based materials [67,135,136]. Additionally, the presence of ultrafine particles (0.1–7%) can further enhance the density of concrete mixtures by filling microvoids and reducing porosity. These fine particles also promote microstructural refinement and nucleation processes, leading to improved performance in materials such as cementitious composites, radiation-shielding materials, and glass-based systems [137].

Several physical and mechanical tests are commonly employed to evaluate the quality and performance of wood ash (WA)-based concretes, including setting time, slump flow, compressive strength, tensile strength, flexural strength, density, water absorption, acid resistance, and microstructural analysis. These parameters provide critical insights into the workability, mechanical behaviour, and durability of WA-modified cementitious systems. The incorporation of WA as a partial replacement for cement generally influences the hydration kinetics of concrete, often resulting in a delay in the setting time of the mixture. This delay is primarily attributed to the presence of higher contents of CaO, alkali oxides, and loss on ignition (LOI) in the ash, which can alter the hydration reactions and early-age microstructure of the cement matrix. Experimental studies have shown that concrete containing approximately 10% WA replacement may exhibit a relatively moderate or even slightly reduced setting time compared with higher replacement levels. However, as the proportion of WA increases beyond this threshold, the setting time tends to increase significantly, which can affect the early-age performance and workability of the concrete mixture [60,138,139,140,141,142,143].

## 5. Computational Analysis of Wood Ash Novel Materials

Computational modelling and data-driven approaches have recently emerged as powerful tools for the prediction and optimization of mechanical behaviour and industrial applications of wood ash (WA)-based materials [141]. Advanced soft computing and machine learning techniques enable the analysis of complex relationships between the physical, chemical, and mineralogical characteristics of WA and the resulting performance of engineered materials. For instance, Chowdhury et al. employed soft computing models to evaluate the properties of WA and successfully predicted key strength parameters including compressive, split tensile, and flexural strengths using Support Vector Machine (SVM) algorithms [39]. Similarly, Rohan et al. investigated alkali-activated systems derived from fly ash and demonstrated that Random Forest (RF) models can effectively predict mechanical, microstructural, and structural properties, as well as key processing parameters involved in material synthesis [144]. More broadly, machine learning-driven artificial intelligence (AI) approaches provide an efficient framework for identifying hidden patterns within large experimental datasets, enabling improved prediction accuracy and optimization of material design. These computational techniques can also help mitigate uncertainties and limitations associated with traditional analytical models by detecting defects, processing variations, and performance-related shortcomings in WA-based materials [145], as summarized in Table 2.

Similar computational and numerical approaches have also been applied to the analysis of wood ash (WA)-based composites and glass systems. For instance, Sarmad et al. conducted both experimental and numerical investigations on WA-reinforced polyester and fibreglass composites, focusing on their processing conditions and mechanical performance. In their study, finite element modelling using ANSYS software was employed to simulate and evaluate the mechanical behaviour of WA-based polyester and fibreglass composite structures, demonstrating the capability of numerical tools for predicting material performance and optimizing composite design [146]. Likewise, Gharam et al. and Külekçi et al. reported similar research on the development, characterization, and testing of WA-derived glass systems and high-energy radiation-shielding concretes, highlighting the importance of computational modelling in understanding their structural and protective properties [147,148]. In recent years, artificial intelligence (AI)-driven automation and digital manufacturing technologies have increasingly begun to replace conventional manufacturing approaches in advanced materials engineering [19,149]. The integration of AI-based modelling, automated processing, and digitalization techniques offers a powerful framework for improving process control, material optimization, and production efficiency in WA-based material systems. Consequently, these emerging technologies can serve as effective tools for enhancing the sustainability, resource efficiency, and industrial scalability of wood ash-derived products, as summarized in Table 2.

Despite the growing interest in AI-assisted materials design, several challenges still limit the widespread implementation of machine learning models for wood ash-based materials. The heterogeneity of wood ash composition, limited availability of standardized datasets, and variations in processing conditions often reduce the generalization capabilities of predictive models. In addition, black-box algorithms may suffer from poor interpretability, limiting their acceptance in industrial applications. Consequently, explainable artificial intelligence (XAI) approaches and uncertainty quantification methods are becoming increasingly important for improving the reliability and transparency of predictive models. Looking forward, the integration of physics-informed machine learning, digital twins, autonomous experimentation, and inverse materials design is expected to accelerate the development of high-performance and sustainable wood ash-derived materials. Coupling AI with Industry 4.0 technologies, sensor-based monitoring, and digital manufacturing platforms may enable real-time process optimization and intelligent quality control. Furthermore, the development of FAIR (Findable, Accessible, Interoperable, and Reusable) databases and hybrid modelling frameworks combining experimental data with computational simulations will facilitate the transition from laboratory-scale investigations to industrial-scale implementation. Therefore, AI and machine learning are expected to evolve from predictive tools toward intelligent decision-support systems for sustainable circular manufacturing [151,152,153].

## 6. Quality Control and Assurance for Industrialization

Laboratory-based inspection and testing procedures play a crucial role in evaluating the performance, reliability, and quality of manufactured wood ash (WA)-based materials. To ensure consistency and reproducibility, several international standards such as those developed by the American Society for Testing and Materials (ASTM), Canadian Standards Association (CSA), and European Standards (EN) are widely employed to characterize the physical, chemical, thermal, and surface properties of WA-derived materials. These standardized procedures enable the systematic assessment of key parameters including material extraction, free expansion, waste evaluation, bending strength, and tensile behaviour, as summarized in Table 3. Furthermore, quality control (QC) and quality assurance (QA) are essential components of the evaluation framework for WA-based products. QC procedures are typically implemented during material processing and manufacturing stages, including recycling and formulation processes, to monitor material consistency and detect defects. In contrast, QA procedures are conducted after production to verify that the final products meet the required performance and safety standards. Together, QC and QA methodologies provide a comprehensive evaluation of the physical and chemical behaviour of WA-based materials, ensuring their suitability for engineering and industrial applications [68,150,154], as presented in Table 3.

Controlled combustion of wood biomass plays a critical role in producing wood ash (WA) with tailored physicochemical characteristics that meet specific application requirements [68,155]. By regulating combustion conditions such as temperature, oxygen availability, and residence time, it is possible to influence the composition, particle morphology, and mineral phases of the resulting ash. Following combustion, WA typically undergoes initial physical, chemical, thermal, and spectroscopic characterization, which provides essential information for determining suitable recycling routes, manufacturing processes, and potential applications of WA-derived materials [70,150,151,152,153,154,155,156,157,158,159,160,161]. Subsequently, WA can be transformed into a variety of value-added products—including cementitious concretes, glass systems, and polymer or ceramic composites—through different recycling and manufacturing techniques. These material transformations are carried out at both laboratory and industrial scales using standardized processing methods and quality protocols [162,163,164]. Among the various evaluation procedures, the compressive strength test of concrete remains one of the most important parameters for assessing the mechanical performance and structural integrity of WA-based construction materials [165], as summarized in Table 3.

Wood ash (WA)-based polymeric and ceramic composites require comprehensive mechanical characterization after manufacturing to evaluate their structural performance and reliability. Important mechanical properties such as tensile strength, elastic modulus, yield strength, toughness, brittleness, compressive strength, fracture behaviour, and impact energy absorption are commonly assessed through standardized mechanical testing procedures [166,167,168]. In addition, tribological investigations are conducted to examine surface-related characteristics, including wear resistance, friction behaviour, and surface durability of WA-based composites. Hardness measurements are also performed to determine the material’s resistance to localized deformation and surface damage. These mechanical and tribological evaluations provide critical information regarding the strength, durability, and functional performance of WA-derived polymeric and ceramic composite materials for engineering applications [169,170,171,172,173,174,175].

**Table 3:** Quality control and quality assurance of wood ash materials.

Analysis Type	Testing Methods		Outcomes	Reference
	At Laboratory Scale	At Industrial Scale		
Heating of WA	Test Methods D3682, D4326, and D6349, D3683, D6357	In accordance with internationally recognized principles of standardization. D3174 and D7582	Burning of agriculture, residue and WA in different environments.	[150]
Extraction of wood ash	ASTM D3987-81	ASTM D3987-12 (2020)	Leaching of wood ash and extraction of minerals	[68]
Free expansion of the ash	ASTM (D1883)	ASTM C618, ASTM C311, Autoclave test (ASTM C151), (ASTM D1883)	Assessment of potential for expansion caused by hydration of calcium oxide and magnesium oxide	[68,151]
Wood waste evaluation	Canadian Standards Association (CSA) A23.5, ASTM C618	Canadian Standards Association (CSA) A23.5, ASTM C618	WA is categorized into Type F (CaO ≤ 8%), Type Cl (CaO ≤ 20%), and Type CH (CaO > 20%)	[96]
Chemical composition of WA	EN ISO 16967:2015	EN ISO 16967:2015	Testing procedure for WA according to The European Committee for Standardization, 2015a	[152]
Loss of ignition	*ASTM D 7348-13*	ASTM D7348-21, ASTM D 7348-13 (ASTM International, 2013)	Determination of LOI of WA under different combustion conditions	[153]
Contents of carbon, hydrogen, and nitrogen in WA	*EN ISO 16948:2015*	*EN ISO 16948:2015-07*	Determination of hydrogen and nitrogen content in WA	[154]
Pozzolanic activity	Modified Chapelle test NF P18-513	BS 3892, ASTM C125, EN 196-5 and ASTM C311	Testing of *siliceous and aluminous materials*	[155]
Density of WA	ASTM C-188 (ASTM International, 2017) and EN 1097-6:2013 (The European Committee for Standardization, 2013a)	Same as laboratory testing	Determination of density of wood bottom and fly ash	[156,157,158,159,160,161]
Morphologies, particle size, composition	Spectroscopic analysis according to standard operating procedures (SOPs)	XRD, SEM, TEM, etc., according to standard operating procedures	Evaluation of physical properties of WA	[67]
Green manufacturing of concrete and concrete structures	ASTM A82–ASTM A 775 series for steel, plain and deformed concretes ASTM C 14–ASTM C 1603 series for concretes, aggregates, blends, mixtures, polymeric composites, mortars. ASTM D 1248–ASTM D 2240 series for plastics and rubbery materials	ISO 13.315-1 (2012) and ISO 13.315-2 (2014), ASTM A82–ASTM A 775 series for steel, plain and deformed concretes, ASTM C 14–ASTM C 1603 series for concretes, aggregates, blends, mixtures, polymeric composites, mortars. ASTM D 1248–ASTM D 2240 series for plastics and rubbery materials	Industrial and green manufacturing of concrete, including life cycle assessment	[161,162,163,164,165,166]
Compressive strength of concrete	ASTM C39/C39M-18	ASTM C39/C39M-21	Evaluation of compressive strength of cylindrical concrete specimens	[170]
Wood ash polymeric and ceramic composites	ASTM D3039 (tensile), ASTM D5467 (bending), and ASTM A370 (impact testing) for polymers. ASTM C1273-18, ASTM C1341-13 (2018), and ASTM D7136/D7136M-12 for ceramic materials	Same as laboratory testing of polymers.	Evaluation of tensile, bending and impact properties of polymeric and ceramic composites	[171,172]
Wood ash-based glass systems	ASTM C158-02 (bending), ASTM C158 (tensile testing), and BS EN 12600 (impact testing)	Same as laboratory testing	Determination of tensile, impact, and bending properties of novel wood ash glass systems	[173,174,175]
Gamma radiation shielding performance of wood ash-based glass systems	ASTM C1831/C1831M-17	Same as laboratory testing	Evaluation of gamma radiation shielding performance	[172,173,174,175,176]

## 7. Wood Ash Novel Product Materials and Customer Satisfaction

Quality assurance (QA), which largely relies on the outcomes of quality control (QC) procedures, represents an extended framework for the inspection and evaluation of wood ash (WA)-based materials. The utilization of wood bottom ash (WBA) and wood fly ash (WFA) in the manufacturing of concrete and its by-products, glass systems, polymeric composites, and ceramic materials significantly contributes to sustainability by reducing WA waste, lowering carbon emissions, and promoting circular and closed-loop manufacturing processes. QC procedures, together with inspection and testing methods, are employed to assess the mechanical performance and material characteristics of WA-derived products. Subsequently, QA acts as a comprehensive evaluation tool to determine the quality and purity levels of WA, which may be classified into low, medium, and high grades depending on compositional and performance criteria. The appropriate grade of WA is therefore selected according to the processing technique, material formulation, and intended application of the final product. Ultimately, QA represents the final stage of performance verification, ensuring that WA-based materials meet the required technical standards before their utilization by end users and industrial customers.

**Figure 7:**
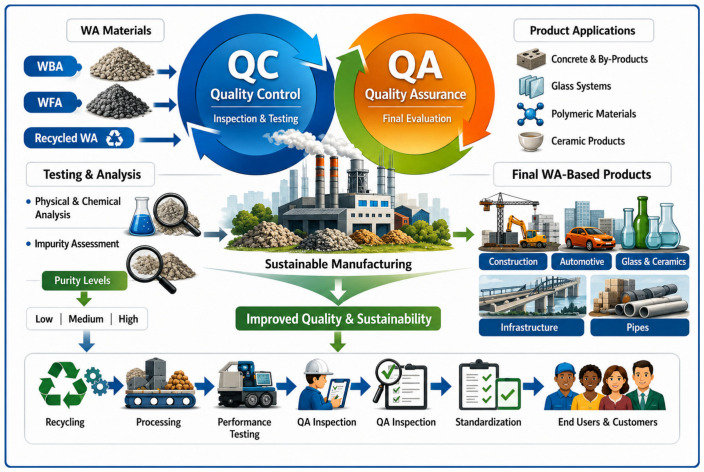
Quality assurance of WA-based materials. Generated by ChatGPT (OpenAI, GPT-5.5).

The testing procedures applied to wood ash (WA)-based materials are largely determined by the requirements of end users, potential commercial applications, and the intrinsic properties of the WA-derived products. The presence of impurities or compositional variations in WA can significantly influence the processing behaviour, microstructure, and final quality of manufactured materials. Therefore, the implementation of quality assurance (QA) frameworks is essential to refine testing methodologies and improve the overall reliability of processing techniques for WA-based novel materials. In parallel, quality control (QC) procedures and standardized testing protocols are continuously improved to ensure consistent evaluation of material properties. Following recycling and comprehensive physical and chemical characterization, WA-derived products are supplied to end users and industrial customers. Through systematic QC and QA practices, important parameters such as service life, performance, usability, workability, and product diversity can be effectively standardized. Consequently, these integrated quality management systems facilitate the industrialization and commercialization of WA-based materials, enabling their broader adoption in sustainable engineering applications, as illustrated in Figure 7.

## 8. Costing of Additive Manufacturing Process and Wood Ash Novel Products

Cost analysis is an important step in evaluating the economic feasibility of material processing during research and development activities. It provides a systematic approach to estimating the economic viability and scalability of manufacturing processes for WA-based materials. In particular, economic assessment helps determine the cost implications associated with raw materials, processing techniques, energy consumption, and production scale. The cradle-to-cradle (CTC) concept is frequently employed to evaluate the overall cost and sustainability of materials throughout their lifecycle, including homogeneous, continuous, batch, and heterogeneous production systems. For example, Hussain et al. proposed a break-even analysis model to evaluate the cost structure of WA-based material production and to determine the production level at which revenues equal total costs. Furthermore, order costing and process costing approaches are commonly adopted to link manufacturing economics with customer requirements and specific production systems, enabling more effective economic planning and industrial implementation.

The breakdown of total production expenses into individual cost components is referred to as cost evaluation. This process enables a systematic assessment of the economic factors involved in material processing and manufacturing. Table 4 summarizes the different cost elements and corresponding analytical approaches used in the evaluation process. Cost assessment, pricing strategies, and the interaction between manufacturers and customers play a crucial role in determining the economic viability and commercial success of manufacturing operations. In particular, the selling price of a product influences the balance between market demand and production capacity, thereby guiding economic decision-making in industrial processes. Consequently, these economic evaluation frameworks can be effectively applied to determine the cost feasibility and commercial potential of wood ash (WA)-based novel materials.

**Figure 8:**
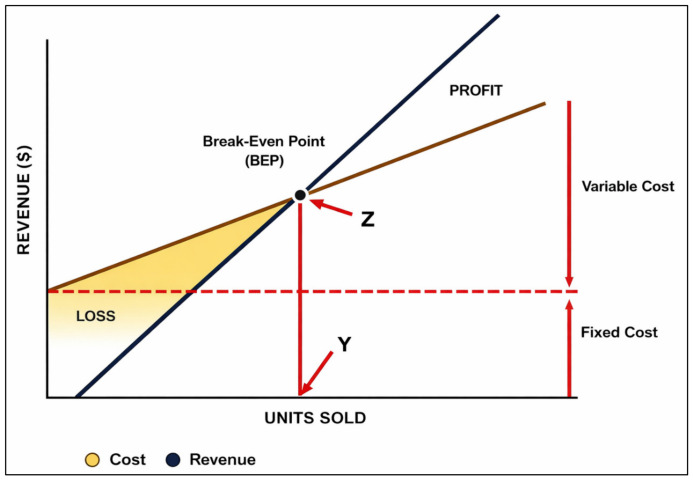
Costing and BEA model.

Break-even analysis (BEA) is an important economic evaluation tool used to assess the commercial feasibility and operational sustainability of manufacturing systems. As illustrated in Figure 8, the sales of wood ash (WA)-based products can be analyzed as a function of revenue generation and profit margins, which collectively determine the financial performance of the production system. BEA provides a quantitative framework for identifying the production level at which total revenues equal total costs, thereby indicating the point at which a manufacturing process transitions from loss to profit. In the present context, the theoretical implementation of BEA is considered for evaluating the economic viability of WA-based material production. The mathematical formulation of the break-even condition can be expressed as follows:

$$Total\;Cost\;\left(TC\right)\;of\;WA\;products=Fixed\;Cost\;\left(FC\right)\;of\;WA\;Products+Variable\;Cost\left(VC\right)of\;WA\;products$$

Total cost (TC) plays a fundamental role in evaluating the economic feasibility and initiation of a wood ash (WA)-based manufacturing system. It comprises both fixed costs (FC) and variable costs (VC) that are associated with the production process. Fixed cost represents the initial investment required to establish the manufacturing infrastructure, including equipment, facilities, and operational setup, and generally remains constant regardless of production volume. In contrast, variable cost is directly associated with the processing and production of WA-based materials and varies according to the quantity of units produced. Consequently, changes in VC can significantly influence the profit margins and revenue generation of the manufacturing system. The break-even point (BEP) is defined as the stage at which the total revenue generated from the sale of WA-based products is equal to the total production cost, resulting in neither profit nor loss. Analysis of BEA and variations in BEP can therefore provide valuable insights into the initiation, expansion, decline, and growth potential of WA-based product manufacturing systems, as illustrated in Figure 8 and Table 4.

## 9. Innovative Circularity Model and Sustainability of Novel Wood Ash Products

The concept of circularity refers to an industrial system based on cradle-to-cradle (closed-loop) manufacturing, sustainable production systems, and performance-oriented economic models. Within this framework, waste materials are continuously reintegrated into production cycles to enhance resource efficiency and environmental sustainability. Recent research has advanced the understanding of wood ash (WA) characterization, circular manufacturing pathways, quality assurance (QA), quality control (QC), and economic evaluation, and aims to improve the sustainability and industrial utilization of WA-based materials. Detailed investigations of wood combustion processes together with physical, chemical, and thermal characterization of WA help determine the purity level, appropriate manufacturing routes, and potential commercial applications of WA-derived products. Based on compositional analysis, WA can generally be categorized into low-, medium-, and high-purity grades, each suitable for different industrial applications. The global production of WA is estimated to be approximately 18.5 million tonnes annually, while Europe contributes around 2.42 million tonnes [1,2]. High-purity WA can be effectively used as a functional filler or additive in polymeric and ceramic composites, whereas another promising application includes the production of glass systems, particularly when the silica content exceeds approximately 63%. Additionally, WA containing more than 70% pozzolanic oxides is suitable for the recycling and manufacturing of cement-based materials such as concrete. Consequently, the valorisation of WA as a secondary raw material enables the production of a wide range of sustainable products. This approach contributes to reducing manufacturing costs, minimizing environmental impacts, lowering greenhouse gas emissions, decreasing waste accumulation, and preserving natural resources, thereby supporting the broader goals of circular economy and sustainable materials development.

**Figure 9:**
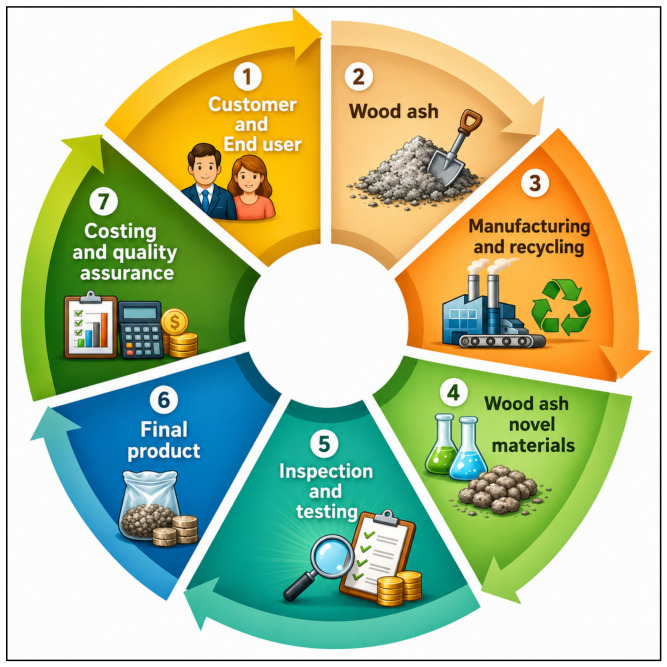
Proposed circularity and commercialization of wood ash materials. Generated by ChatGPT (OpenAI, GPT-5.5).

The transformation of wood ash (WA) into sustainable engineering products remains an active area of research that requires further scientific investigation and technological development. The evaluation and performance assessment of WA-derived materials are closely linked to their potential industrial and commercial applications. As illustrated in Figure 9, the circular and environmentally sustainable production pathway includes several interconnected stages such as material characterization, recycling and manufacturing processes, quality control (QC), quality assurance (QA), economic evaluation, and commercialization strategies. The performance evaluation of WA-based products is essential for determining their service life, structural integrity, durability, and overall product quality, which ultimately influence their acceptance in industrial and construction sectors. In addition, the implementation of internationally recognized standards including ISO, ASTM, CSA, and European standards plays a crucial role in enabling the industrial production, certification, and commercialization of WA-based materials. These standards provide systematic procedures for testing mechanical properties, durability, chemical stability, and environmental compatibility of WA-derived products. The conversion of WA waste into value-added materials such as cementitious concretes, glass systems, polymer composites, and ceramic materials significantly enhances the overall sustainability of manufacturing systems by promoting resource recovery and waste valorisation. From a broader sustainability perspective, the utilization of WA-based products contributes simultaneously to environmental, economic, technical, and social dimensions of sustainable development. Environmentally, it reduces landfill disposal of ash residues and lowers greenhouse gas emissions associated with conventional material production. Economically, the incorporation of WA as a secondary raw material decreases production costs and improves resource efficiency. Technically, WA-derived materials provide promising functional properties such as enhanced mechanical performance, thermal stability, and radiation shielding capability. Socially, the implementation of circular manufacturing systems promotes sustainable industrial practices and supports the transition toward low-carbon economies. Furthermore, the integration of cost analysis, break-even evaluation, and quality assurance strategies facilitates the development of viable business models for WA-based materials, ensuring both economic feasibility and industrial competitiveness. Through systematic testing, certification, and economic assessment, manufacturers can improve product reliability and meet the expectations of customers, end users, and regulatory authorities. Consequently, the adoption of circular manufacturing strategies for WA valorisation not only enhances sustainability but also supports the large-scale industrial deployment and commercialization of innovative WA-based materials. From an industrial perspective, the applicability of WA-derived materials is governed not only by mechanical performance but also by raw material variability, processability, quality assurance requirements, cost effectiveness, and regulatory constraints. Therefore, the practical performance boundaries of WA-based materials are application-dependent and require optimization through standardized characterization, process control, and techno-economic evaluation. These factors are essential for successful large-scale commercialization and industrial implementation.

## 10. Future Research Directions

Despite the significant progress achieved in the valorisation of wood ash (WA) for sustainable materials, several research challenges and opportunities remain that require further investigation. The following directions may guide future research and industrial development of WA-based materials:

  i. **Standardization of wood ash characterization and classification:** Future research should focus on establishing globally standardized protocols for the characterization and classification of WA, considering variations in biomass sources, combustion technologies, and ash collection systems. Developing unified classification systems based on chemical composition, particle morphology, and pozzolanic activity will improve consistency in research outcomes and facilitate the industrial adoption of WA-derived materials.
 ii. **Advanced manufacturing technologies for WA-based materials:** Further studies are required to explore innovative manufacturing technologies, including additive manufacturing (3D printing), geopolymer synthesis, and hybrid composite fabrication, for converting WA into high-performance materials. Optimization of processing parameters such as particle size distribution, binder composition, and curing conditions will be essential for achieving improved mechanical performance and structural reliability.
iii. **Integration of artificial intelligence and digital manufacturing systems:** The application of machine learning, artificial intelligence, and digital twin technologies should be expanded to predict material behaviour, optimize processing parameters, and improve automation in WA-based manufacturing systems. AI-driven approaches can accelerate material design, reduce experimental costs, and support Industry 4.0 and Industry 5.0 integration in sustainable materials processing.
 iv. **Life-cycle assessment and environmental impact evaluation:** Comprehensive life-cycle assessment (LCA) studies are necessary to evaluate the environmental performance of WA-based products throughout their lifecycle. Future investigations should quantify carbon footprint reduction, energy savings, and resource efficiency associated with the substitution of conventional materials with WA-derived alternatives.
  v. **Long-term durability and performance evaluation:** Although several studies have demonstrated the mechanical potential of WA-based materials, further research is needed to investigate their long-term durability, chemical resistance, and structural stability under real service conditions. This includes studies on corrosion resistance, freeze–thaw durability, sulphate attack, and radiation shielding performance for infrastructure and industrial applications.
 vi. **Industrial scalability and commercialization strategies:** Future research should also address the economic feasibility and industrial scalability of WA valorisation technologies. This includes the development of cost-modelling frameworks, break-even analysis, and supply chain optimization to support large-scale commercialization. Collaboration between academia, industry, and policymakers will be essential to integrate WA-based materials into circular manufacturing systems and sustainable construction practices.

## 11. Conclusions

This review presents a comprehensive technical pathway for the analysis, manufacturing, characterization, computational modelling, and commercialization of wood ash (WA)-based novel materials within a circular economy framework. The major conclusions of the study are summarized as follows:

   I. **Wood ash composition and chemical characteristics:** The combustion of wood biomass produces ash rich in major mineral oxides including SiO_2_, Al_2_O_3_, CaO, and Fe_2_O_3_, while smaller quantities of MgO, Na_2_O, K_2_O, SO_3_, and Cl are also present. Wood ash generally exhibits a highly alkaline nature, with pH values ranging from 9 to 13.5. At lower temperatures (<500 °C), mineral phases are predominantly present as carbonates and bicarbonates, whereas oxide phases dominate at temperatures exceeding 1000 °C due to thermal decomposition reactions. The combined content of pozzolanic oxides (SiO_2_ + Al_2_O_3_ + Fe_2_O_3_) can serve as an important indicator of WA purity and its suitability for cementitious applications. The overall composition of WA is strongly influenced by factors such as wood species, combustion technology, temperature conditions, biomass origin, and combustion environment.
  II. **Morphology and potential applications:** Wood ash particles typically exhibit angular, spherical, and irregular morphologies, which influence their reactivity and packing behaviour in composite systems. WA can be effectively utilized in the manufacturing of cementitious concretes, glass systems, and polymeric or ceramic composites. In cementitious applications, the presence of pozzolanic oxides exceeding approximately 70% significantly improves the performance of WA-based concrete systems. Similarly, ashes containing SiO_2_ contents greater than about 62% are suitable for the production of glass and glass–ceramic materials.
 III. **Performance of WA in cementitious materials:** The partial replacement of cement with WA represents one of the most promising applications of wood ash in construction materials. Numerous studies indicate that 5–20% replacement of cement with WA can enhance or maintain the mechanical and physical properties of concrete. However, excessive replacement levels may introduce microstructural defects such as increased porosity, voids, and surface irregularities, which can reduce structural integrity. In some cases, higher replacement levels (20–80%) may still improve mechanical properties depending on ash composition, mineral content, and combustion conditions, highlighting the importance of optimized material design.
  IV. **Advanced material applications:** Beyond cementitious materials, WA can also be utilized for the production of glass systems, fillers, and additives in polymeric and ceramic composites. In particular, wood fly ash and agricultural ashes with high silica content can serve as valuable precursors for radiation-shielding glasses and functional composite materials, offering improved thermal stability and mechanical performance.
   V. **Inspection, testing, and standardization:** The inspection and testing procedures for WA-based materials depend on the intended product and application. Comprehensive physical, chemical, thermal, and spectroscopic characterization of WA is essential for determining its suitability for different manufacturing processes. Standardized quality control (QC) and quality assurance (QA) protocols ensure the reliability and performance of WA-based materials, including concrete, glass systems, and polymeric or ceramic composites, throughout the manufacturing lifecycle.
  VI. **Computational modelling and digital manufacturing:** Recent studies have increasingly incorporated computational modelling and artificial intelligence techniques to predict the behaviour of WA-based materials. Tools such as Support Vector Machines (SVM), Random Forest (RF), deep neural networks (DNN), artificial neural networks (ANN), Mamdani fuzzy logic (MFL), hybrid neural fuzzy inference systems (HYFIS), FLUKA simulations, and XCOM computations have been successfully applied to predict mechanical performance, radiation shielding properties, and processing parameters. These digital tools also support automation and optimization of modern manufacturing systems.
 VII. **Economic evaluation and industrial feasibility:** Economic assessment methods such as break-even analysis (BEA) and break-even point (BEP) play an important role in evaluating the commercial feasibility of WA-based manufacturing systems. These models consider total cost, fixed cost, and variable cost components to determine the profitability and scalability of WA product manufacturing. Such economic tools provide insights into the initiation, expansion, and long-term sustainability of WA-based industrial processes.
VIII. **Circular economy and sustainability:** The integration of circular economy principles significantly enhances the sustainability potential of WA-based materials. The valorisation of WA for the production of concrete, glass systems, and composite materials promotes closed-loop manufacturing systems, reduces industrial waste, lowers greenhouse gas emissions, and improves resource efficiency. Consequently, WA can serve as an important secondary raw material for sustainable manufacturing, supporting environmentally responsible production systems and advancing circular industrial development.

## 12. Materials and Methods

Generative AI-assisted technology was used to support scientific writing and organization of the manuscript. The tool was employed to assist in drafting and refining text, while all experimental design, data analysis, interpretation, and conclusions were independently performed and verified by the authors.

## Figures and Tables

**Table 2:** Computation of wood ash-based products.

Testing Method	Tested Properties	Research Outcomes	Reference
Support Vector Machine (SVM) algorithms	Strength parameters like compressive, split tensile, and flexural	Successfully used SVM to predict the Strength parameters	[35,141]
RF machine learning studies	mechanical, microstructural, structural properties and processing parameters	Prediction of physical parameters of mechanical properties and processing	[144]
Deep neural network (DNN) artificial neural network	Compressive strength of green fly ash-based geopolymer concrete	Prediction of compressive strength of fly ash-based geopolymer concretes using DNN and ResNet machine learning approaches	[145]
ANSYS	experimental and numerical investigations of WA-based polyester and fibreglass composites.	Prediction of mechanical behaviour and processing of composites	[146]
FLUKA simulations and XCOM computation	Photon mass attenuation coefficient of the glasses was computed	Investigation of glass systems for their radiation-absorbing ability	[147]
Linear attenuation coefficient calculations	Examination of gamma radiation absorption properties	Investigation of gamma ray properties of lightweight WA-based concretes for production	[148]
Python-based AI automation and digitalization. Random forest (RF), artificial neural network (ANN), Mamdani fuzzy logic (MFL), hybrid neural fuzzy inference system (HYFIS)	Process automation, digitalization	Automation of processing	[19,149,150]

**Table 4:** Functions of various costs.

Cost Type	Cost Functions
Variable cost	Labour cost, raw materials cost, machinery cost, packaging cost, other royalties
Fixed cost	Depreciation, rent, office salaries
Total cost	Fixed cost, variable cost
Direct costs	Variable costs, supplier costs
Indirect costs	Administration costs, distribution costs, selling costs
Factory overheads	Taxes, utilities costs, management costs, depreciations

## Data Availability

No new data were created or analyzed in this study. Data sharing is not applicable to this article.

## Abbreviations

WA	Wood ash	pH	Potential of hydrogen
µm	Micrometre	%	Percentage
QC	Quality control	QA	Quality assurance
ASTM	American Society for Testing and Materials	CSA	Canadian Standards Association
Gt	Gigatons	Mt	Million tonnes
WBA	Wood bottom ash	WFA	Wood fly ash
CE	Circular economy	3DP	Three-dimensional printing
EN	European Standard	DSC	Differential scanning calorimetry
TGA	Thermogravimetric analysis	SEM	Scanning electron microscopy
AI	Artificial intelligence	ANOVA	Analysis of variance
BEA	Break-even analysis	SiO_2_	Silicon dioxide
CaO	Calcium oxide	Al_2_O_3_	Aluminium oxide
Fe_2_O_3_	Iron(III) oxide	K_2_O	Potassium oxide
MgO	Magnesium oxide	O_2_	Oxide
CaCO_3_	Calcium carbonate	CO_2_	Carbon dioxide
H_2_O	Water	Ca(OH)_2_	Calcium hydroxide
Ca(HCO_3_)_2_	Calcium bicarbonate	°C	Degrees Celsius
Na	Sodium	K	Potassium
m	Meter	g	Gram
cm	Centimetre	P_2_O_5_	phosphorus pentoxide
SO_3_	Sulfur trioxide	L**OI**	Loss on ignition
wt.	Weight	F_2_O_2_	Fluorine peroxide
TiO_2_	Titanium dioxide	AM	Additive manufacturing
CS	Compressive strength	Pb	Lead
Bi	Bismuth	W	Tungsten
ZnO	Zinc oxide	SrO	Strontium oxide
ZrO_2_	Zirconium dioxide	BaO	Barium oxide
SVM	Support Vector Machine	RF	Random Forest
DNN	Deep neural network	ANN	Artificial neural network
XAI	Explainable artificial intelligence	MFL	Mamdani fuzzy logic
HYFIS	Hybrid neural fuzzy inference system	ISO	The International Organization for Standardization
SOPs	Standard operating procedures	CTC	Cradle-to-cradle
TC	Total cost	FC	Fixed cost
VC	Variable cost	BEP	Break-even point
